# Multidetector CT imaging of complications after laparoscopic nephron-sparing surgery

**DOI:** 10.1007/s13244-015-0413-1

**Published:** 2015-06-24

**Authors:** Massimo Tonolini, Anna Maria Ierardi, Virginia Varca, Giacomo Piero Incarbone, Marina Petullà, Roberto Bianco

**Affiliations:** Department of Radiology, “Luigi Sacco” University Hospital, Via G.B. Grassi 74, 20157 Milan, Italy; Interventional Radiology, Department of Radiology, University of Insubria, Viale Borri 57, 21100 Varese, Italy; Department of Urology, “Luigi Sacco” University Hospital, Via G.B. Grassi 74, 20157 Milan, Italy

**Keywords:** Laparoscopic nephron-sparing surgery, Haemorrhage, Pseudoaneurysm, Urine leak, Computed tomography (CT)

## Abstract

**Purpose:**

Laparoscopic nephron-sparing surgery (L-NSS) is increasingly performed to treat localised renal lesions. However, the associated morbidity is non-negligible, with a rate of major complications approaching 10 %.

**Methods and Results:**

This paper provides an overview of indications, surgical techniques and results of L-NSS; explains the incidence, risk factors and manifestations of postoperative complications; discusses the preferred multidetector computed tomography (CT) acquisition techniques; illustrates the appearance of normal postoperative images following L-NSS; and reviews, with example images, the most common and unusual iatrogenic complications. These include haematuria, haemorrhage, vascular injuries, infections and urinary leaks. Most emphasis is placed on CT, which provides rapid, reliable triage and follow-up of iatrogenic complications after L-NSS, identifying occurrences that require transarterial embolisation or repeated surgery.

**Conclusions:**

Multidetector CT allows precise assessment of the surgical resection site; detection of pneumoperitoneum and subcutaneous emphysema; quantification of retroperitoneal blood; and identification of active bleeding, pseudoaneurysms, arterio-venous fistulas, abscess collections and extravasated urine.

***Teaching Points*:**

• *Laparoscopic nephron-sparing surgery (NSS) is increasingly performed to treat renal lesions.*

• *Radiologists are increasingly requested to investigate suspected post-surgical NSS complications.*

• *Post-NSS complications include haemorrhage, haematuria, vascular injuries, infections and urinary leaks.*

• *Multidetector CT allows choice between conservative treatment, transarterial embolisation or surgery.*

## Introduction

### Background

As a result of improved surgical techniques and greater focus on minimising functional impairment over the last decade, the therapeutic approach to localised renal cell carcinoma (RCC) has evolved from the classical radical nephrectomy (RN) towards nephron-sparing surgery (NSS), which was initially performed using an open surgical approach. Compared with RN, open partial nephrectomy (O-PN) achieved a lower rate of postoperative loss of renal function, after adjustment for age, hypertension and diabetes, and similar long-term oncological and quality-of-life outcomes [1, 2].

Meanwhile, the widespread use of ultrasound, computed tomography (CT) and magnetic resonance imaging (MRI) has led to a steady increase in the detection of benign, malignant or indeterminate renal lesions requiring surgery, so that currently almost 50 % of RCCs are diagnosed incidentally, often during imaging studies requested for unrelated reasons. As a result, conservative surgery is increasingly performed to treat patients with small-sized renal masses. According to the most recent guidelines from the European Association of Urology (EAU) [1, 2], NSS represents the treatment of choice for localised RCC. It can be performed with an open, laparoscopic or robot-assisted approach, based on the surgeon’s expertise and skills. Indications for NSS include T1a-b stage RCC and selected masses up to 7 cm, unless contraindicated by unfavourable anatomical location of the tumour or general deterioration of the patient’s condition. Absolute indications include tumours in a solitary kidney, impaired renal function and hereditary disorders that predispose to recurrent RCC. Furthermore, laparoscopy is an appealing minimally invasive treatment for indeterminate renal cysts requiring surgery, and benign masses such as angiomyolipoma or oncocytoma. Relative contraindications for laparoscopy include prior surgical procedures (due to the presence of intra-abdominal adhesions), cirrhosis and portal hypertension, marked bowel distension (which increases the risk for bowel injury), ongoing sepsis and cardiopulmonary disease [1–3].

Both O-PN and laparoscopic partial nephrectomy (L-PN) resulted in superior long-term preservation of renal function. After NSS, patients have a much lower (20 %) 3-year probability of developing chronic kidney disease (defined by <60 mL/min per 1.73 m^2^ estimated glomerular filtration rate [e-GFR]) compared with RN (65 %). At 10 years, the cumulative incidence of chronic renal failure is 22.4 % and 11.6 % for the RN and NSS groups, respectively [4, 5].

Comparison between O-PN and L-PN revealed shorter operating time and warm renal ischaemia time with the open approach, and lower blood loss and shorter hospital stay in the laparoscopic group. No differences were reported in long-term impact on renal function (mean E-GFR decrease 4.1 vs 1.1 mL/min), overall postoperative morbidity and mortality, or progression-free and overall survival (91–94 % 5-year cancer-specific survival) [6–16].

However, L-PN is a technically demanding surgical procedure, with a steep learning curve and potentially serious complications particularly in elderly patients with comorbidities. The overall complication rate in a European multi-institutional series was reported to be 23 %, while a worldwide literature review reported a rate of major complications approaching 10 % [8, 9].

Alternatively, localised renal masses may be treated by laparoscopic or imaging-guided ablative techniques. Although no definite conclusions can be drawn from available studies, surgically treated patients show lower local recurrence rate and cancer-specific mortality; therefore the EAU recommends cryoablation and radiofrequency ablation in elderly and/or comorbid patients with limited life expectancy [1, 2, 17]. However, percutaneous cryoablation and radiofrequency ablation were recently shown to be effective in the treatment of T1 stage RCC, offering excellent preservation of kidney function and similar clinical efficacy and oncological outcome (5-year survival exceeding 90 %) compared with surgery, with a limited incidence of major (4.3–5.6 %) and minor complications and no significant differences between the two modalities [18–21].

### Purpose

Owing to the increasing use of laparoscopy, in hospitals with active surgical practices urologists increasingly request imaging studies to assess patients with suspected postoperative complications following L-PN. This paper provides an overview of the indications, results and technical principles of laparoscopic NSS (L-NSS), and describes the postoperative radiological outcome following L-NSS [22–25].

## Laparoscopic nephron-sparing surgery

### Preoperative assessment

**Fig. 1:**
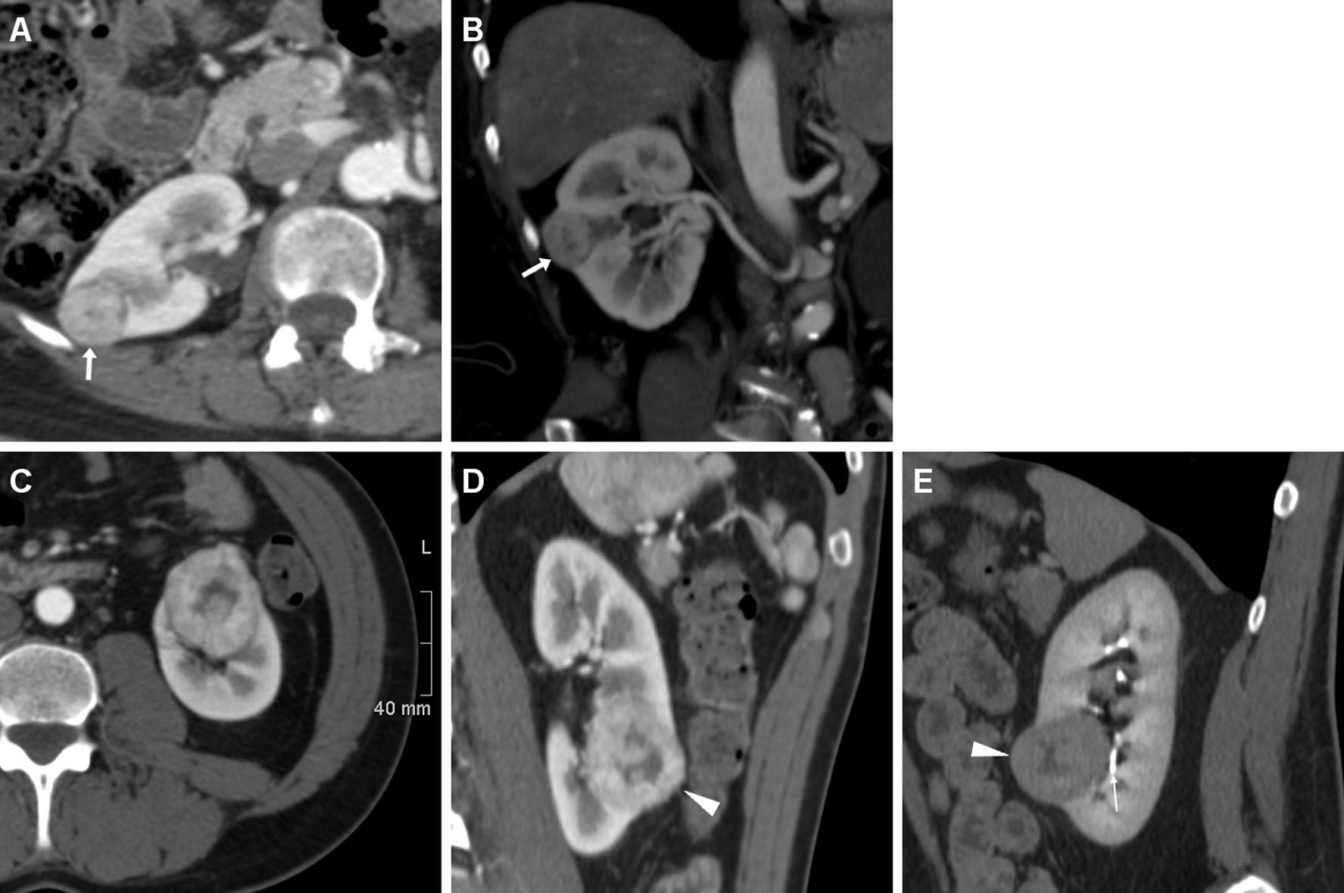
Examples of RENAL nephrometry scores. Total body contrast-enhanced multidetector CT (**a**, **b**) performed for clinical suspicion of systemic lymphoproliferative disease in a healthy 61-year-old woman led to incidental detection of a 2.5-cm, vascularised, partially (<50 %) exophytic mass at the middle third of the right kidney (*arrows*), consistent with T1 stage renal cell carcinoma (RCC). Nephrometry resulted 1 + 2 + 2 + p + 3 (8p). The patient underwent laparoscopic nephron-sparing surgery (L-NSS) complicated by haematoma (*see* Fig. 5). Multiphase CT-urography (**c**–**e**) in a 55-year-old man confirmed ultrasound detection of a 43-mm, inhomogeneously enhancing, left-sided RCC. The lesion appeared partially exophytic, crossed the inferior polar line (**d**) and touched the lower pole calyx (**e**). Nephrometry score was 2 + 2 + 3 + a + 2 (9a). The patient successfully underwent laparoscopic partial nephrectomy (L-PN)

The RENAL (Radius, Exophytic, Nearness, Anterior, Location) nephrometry score (Table 1) was recently introduced as a reproducible means to describe the relevant tumour anatomy, stratify the complexity of renal masses, and objectively compare perioperative and long-term outcomes. The RENAL score includes the five most reproducible features—namely, diameter, exophytic or endophytic growth, proximity to the collecting system, anterior versus posterior location and relationship to the polar lines, which should be assessed on volumetric contrast-enhanced multidetector CT including renal vascular, parenchymal and excretory imaging (Fig. 1) [26, 27]. Tumour staging and complexity dictate the urologist’s therapeutic choice: increasing RENAL scores were found to be strongly associated with RN and O-PN rather than L-NSS [28].

**Table 1:** RENAL nephrometry score (adapted from Parsons et al. [26])

	Score		
Renal lesion feature	1 point	2 points	3 points
R—Radius (maximal diameter)	≤4 cm	4–7 cm	≥7 cm
E—Exophtic vs endophytic	≥50 % Exophytic (projecting outside the renal cortex)	<50 % exophytic	Completely endophytic
N—Nearness to the collecting system/renal sinus	≥7 mm	4–7 mm	≤4 mm
A—Anterior vs posterior location)	Descriptive (no numeric score) “a”, ventral; “p”, dorsal; “x”, others		
L—Location relative to polar lines	Entirely below lower or above upper polar line	Crosses polar line	50 % of mass across polar line Entirely between polar lines Crosses the axial midline
	Additional suffix “h” if tumour reaches hilar vessels		
Renal lesion complexity		RENAL nephrometry score range	
Low		4–6	
Intermediate		7–9	
High		10–12

### Laparoscopic surgical technique

In the majority of patients, L-NSS is performed via the transperitoneal (TP) route; conversely, posterior and postero-lateral renal tumours are best managed with a retroperitoneal approach [9, 11, 29, 30]. Preoperative ureteral catheterisation may be used, particularly when access to the collecting system is necessary. According to some authors, intraoperative ultrasonography for assessment of renal perfusion, tumour location and borders may prove beneficial and result in a change of procedure in a minority (2.5 %) of patients [31]. After laparoscopic access to the retroperitoneum and opening of Gerota’s fascia, en bloc or selective arterial clamping of the renal hilar vascular pedicle is performed to decrease bleeding and ensure a clear surgical field: the acceptable warm ischaemia time is limited to 30 min or less. Depending on the tumour’s size and location, NSS options include segmental polar nephrectomy, wedge resection, transverse resection and enucleation, to obtain complete tumour excision with a proper margin of normal tissue, and preservation of the maximum possible amount of functioning renal parenchyma. After removal of the resected renal portion, surgery requires suture repair of collecting system defects, filling of the parenchymal defect with peri-renal fat or bioabsorbable bolster agents, renal parenchymal reconstruction and closure with a combination of absorbable sutures, argon-beam coagulation and haemostatic agents. Finally, after reconstruction is finished, the vascular clamp is released to restore circulation. Hilar masses, multiple and/or infiltrating tumours with pelvicalyceal involvement pose specific and significant technical challenges for NSS. In selected patients, conversion to laparoscopic RN or O-PN may be necessary when L-NSS is deemed unfeasible by the surgeon after renal exploration [9, 11, 29, 30].

## Postoperative complications: manifestations, incidence and risk factors

The spectrum of non-urological complications after L-NSS includes cardiovascular (deep venous thrombosis, congestive heart failure, atrial fibrillation), pulmonary (pleural effusion, atelectasis/pneumonia, thromboembolism) and gastrointestinal issues (ileus, colonic segmental ischemia, bowel injury, splenic haemorrhage); sepsis; wound infection; and incisional hernias. Specific (urological) complications include massive subcutaneous emphysema, persistent haematuria, haemorrhage, renal vascular injuries, urine leak, renal failure and infections (such as urinary infection, peri-renal abscess and sepsis) [6–12]. Clinically, complications are usually graded by urologists according to the validated Clavien–Dindo system, including, in ascending order of severity, grades I (any deviation from usual postoperative course limited to treatment with anti-emetics, antipyretics, analgesics, diuretics and electrolytes), II (requiring other medical therapies including blood transfusions), III (surgical, endoscopic or interventional treatment), IV (life-threatening complication necessitating intensive care support) and V (death) [32, 33].

Overall, adverse events after L-NSS occur in 23 % of patients: almost two-thirds of cases are minor occurrences (Clavien grades I–II). Despite favourable preoperative patient features and lower objective complexity of tumours, laparoscopic surgery is associated with more major (grade III or higher) overall (6.2-9 % versus 3–6.3 %) and urological (particularly urine leak) complications compared with O-PN [6–12].

The RENAL score is an objective assessment of the complexity of a tumour and may provide a consistent basis for comparing perioperative and long-term outcomes after L-NSS [26, 27]. According to several studies, patients with highly complex tumours are more likely to experience postoperative complications. Posterior location and proximity to the renal sinus seem to have the greatest association with overall complications and haemorrhage [34–39]. Nephrometry scores have been shown to correlate with increased postoperative hospital stay, blood loss, duration of renal ischaemia risk of conversion to open surgery and postoperative renal function loss [35–41].

Conversely, other studies failed to confirm the predictive value of the RENAL score for complications. Apart from tumour size and central growth, other risk factors are reported, including advanced age and comorbidities, limited surgeon’s experience, intraoperative blood loss and opening of the collecting system [40–43].

## Postoperative CT imaging: indications and techniques

Postoperative imaging following L-NSS is generally indicated when clinical features such as hypotension, flank or abdominal pain, gross or persistent haematuria, bleeding from the drainage tube or laboratory abnormalities (particularly blood loss, leucocytosis and increased C-reactive protein levels) suggest a possible complication. Emergency investigation is warranted when signs and symptoms of haemodynamic impairment or sepsis are present [1, 2, 22–25, 29].

In most cases multidetector computed tomography (CT) represents the mainstay imaging technique to comprehensively investigate the abdomen and pelvis in search of possible iatrogenic complications. Experience with blunt body trauma has established that CT is by far the preferred, most rapid and robust technique to depict and grade renal lesions, thus providing the anatomic and functional information necessary for appropriate injury staging and therapeutic choice [22–25, 44]. Intravenous contrast medium (CM) should be administered, unless contraindicated. Since patients that have recently been operated upon are often dehydrated, with limited urine output, the European Society of Urogenital Radiology (ESUR) guidelines recommend special care in ensuring adequate hydration before and after CT, in order to improve urinary tract opacification and to prevent CM nephrotoxicity [45, 46].

In most postoperative urology patients, initial investigation requires a comprehensive multiphase CT acquisition protocol, including: (1) preliminary unenhanced acquisition to demonstrate the postoperative anatomy and detect hyperattenuating blood and abnormal air collections; (2) corticomedullary phase and (3) nephrographic-phase images after CM injection to assess the operated kidney structure and perfusion of the operated kidney, and to identify CM extravasation indicating active bleeding; (4) excretory phase imaging obtained 8–10 min after CM, which demonstrates the opacified urinary cavities and may detect iodinated urine leaks and urinomas. Postoperative CT studies are reviewed interactively on dedicated workstations and complemented with multiplanar reconstructions as necessary, to better depict postoperative anatomy and relevant findings [22–25].

The main drawback of classical multiphase CT protocol is the high radiation dose, which poses a serious concern, particularly considering that these patients usually require serial studies and long-term imaging follow-up. Currently, most institutions are increasingly adopting split-bolus CT-urography acquisitions, which provide combined corticomedullary, nephrographic and excretory imaging with reduced effective radiation dose. In our experience, the time- and dose-efficient triple-bolus protocol described by Kekelidze et al. [47] has proved very useful in the investigation of iatrogenic urinary tract injuries. This technique includes an initial 30-mL CM bolus injected at a flow of 2 mL/s for urinary opacification, followed by a 7-min delay, then a second (50 mL at 1.5 mL/s) CM injection, with a third one (65 mL at 3 mL/s) 20 s later, to provide parenchymal and vascular visualisation respectively, followed by a single volumetric CT acquisition. Alternatively, a combined nephrographic–excretory phase may be obtained by administration of an initial 30–45 mL CM bolus followed, after a 6– to 8-min delay, by a second 75– to 100-mL injection, which may be useful when bleeding or vascular injuries are not suspected, and during follow-up. Furthermore, we recommend repeated (ultra-delayed) excretory acquisition 45–50 min after CM administration in all patients with urinary leak suspected on the basis of surgical, clinical or laboratory data. If available, dual-energy CT may be beneficial to limit the radiation dose, by allowing reconstruction of a virtual unenhanced dataset from CM-enhanced acquisition [47, 48]. Finally, repeated CT provides consistent monitoring of injuries after conservative or interventional treatment [47–49].

## Normal postoperative CT imaging appearances

**Fig. 2:**
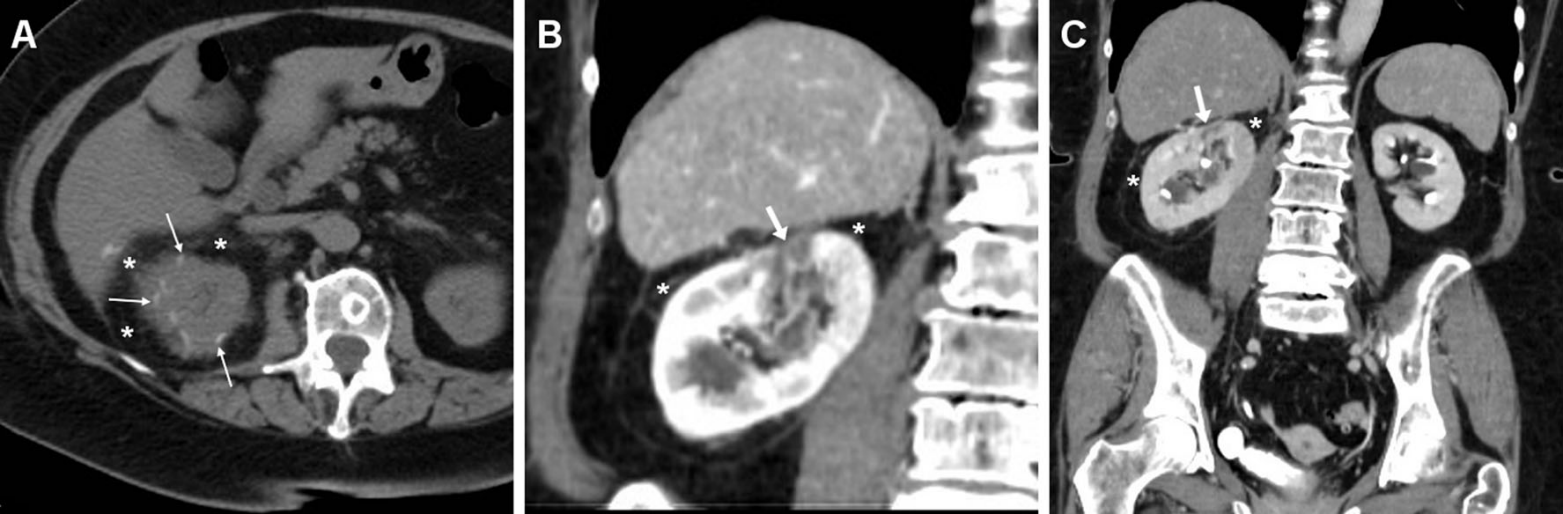
Normal early postoperative imaging appearance in a 77-year-old woman investigated with multidetector CT 4 days after laparoscopic enucleation of a 2-cm RCC of the right kidney. Unenhanced axial image (**a**) showed hyperattenuating linear structures (*thin arrows*) corresponding to surgical sutures at the upper renal pole, and normal peri-renal fat (*). Axial nephrographic (**b**) and coronal excretory-phase (**c**) images showed a focal, wedge-shaped, non-enhancing portion of the renal cortex (*arrows*) with continuous renal contour corresponding to the resection site, and confirmed normal peri-renal fat planes (*) without extravasated blood and urine. Despite postoperative pain and blood loss, the patient had an uneventful postoperative course and was discharged without further treatment

**Fig. 3:**
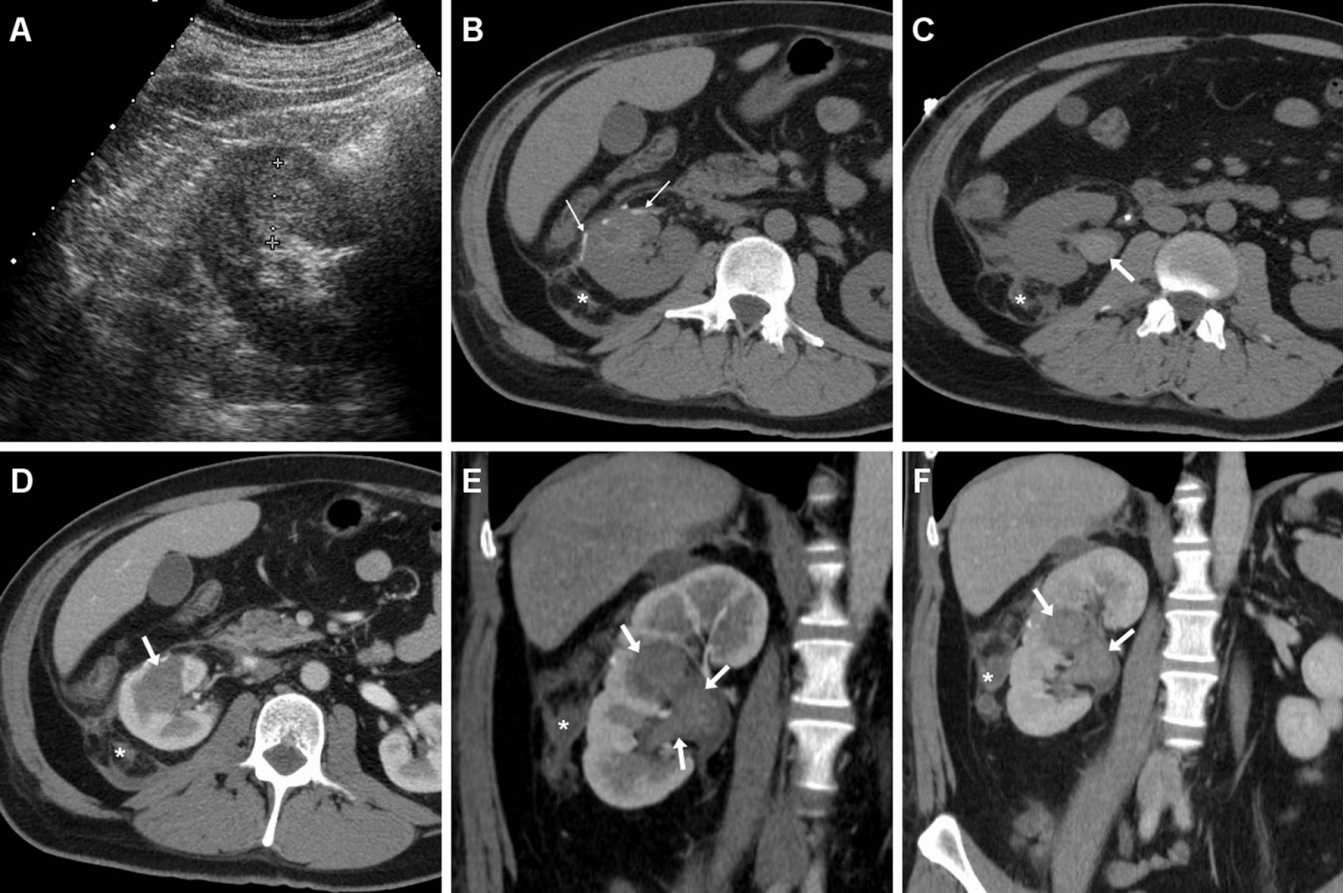
Gross haematuria with hypotension, moderate leucocytosis and blood loss (12 g/dL haemoglobin) in a 32-year-old man shortly after L-PN for a 2.5-cm, hyperechoic RCC (sonographic image in **a**). Unenhanced axial CT images (**a**, **b**) showed hyperattenuating linear structures (*thin arrows*) corresponding to surgical sutures along the ventral renal contour, minimal fluid and blood in the ipsilateral peri-renal space and retroperitoneal fasciae (*), hyperattenuating blood (*arrow*) in the renal pelvis. Corticomedullary (**d**, **e**) and nephrographic (**f**) phase images confirmed blood (*arrows*) occupying most of the renal collecting system. The lack of CM extravasation indicating active bleeding allowed conservative treatment including prolonged vesical catheterisation and lavage

Following tumour resection or limited PN, the surgical site of resection (SSR) may be recognised as a wedge-shaped hypoattenuating portion of the renal cortex, sometimes demarcated by thin linear hyperattenuating sutures (Figs. 2, 3). The SSR generally does not enhance, closely resembles a traumatic laceration and may sometimes tend to shrink or form linear or stellate parenchymal scars in the long-term. After PN, the operated kidney commonly has a more posterior location, and abuts or adheres to the posterior aspect of Gerota’s fascia. Dense thickening of the peri-renal septa, corresponding to fluid or haemorrhagic stranding, is commonly observed in the ipsilateral peri-renal and para-renal spaces (Fig. 3) [22–25].

Sometimes, to improve haemostasis, surgeons may pack the SSR intraoperatively with perinephric fat, which should not be mistaken for a fatty mass. Localised non-enhancing fluid collections corresponding to seroma (sometimes with fat-fluid level) may be visualised. In some patients, biologically absorbable haemostatic agents such as Gelfoam (absorbable gelatine compressed sponge; Pfizer, NY, USA) or Surgicel (oxydised cellulose polymer; Ethicon, Somerville, NJ, USA) may be used to control intraoperative bleeding. Within a few weeks, these bolster agents may show near-water attenuation with interspersed gas foci and can potentially be confused with an abscess; differentiation should rely on knowledge of surgical details, visualisation of gas bubbles arranged in linear fashion and stable appearance or regression on serial scanning. Conversely, abscess should be suspected if a localised fluid collection shows a CM-enhancing rim and contains a gas-fluid level or moving bubbles [22–25].

**Fig. 4:**
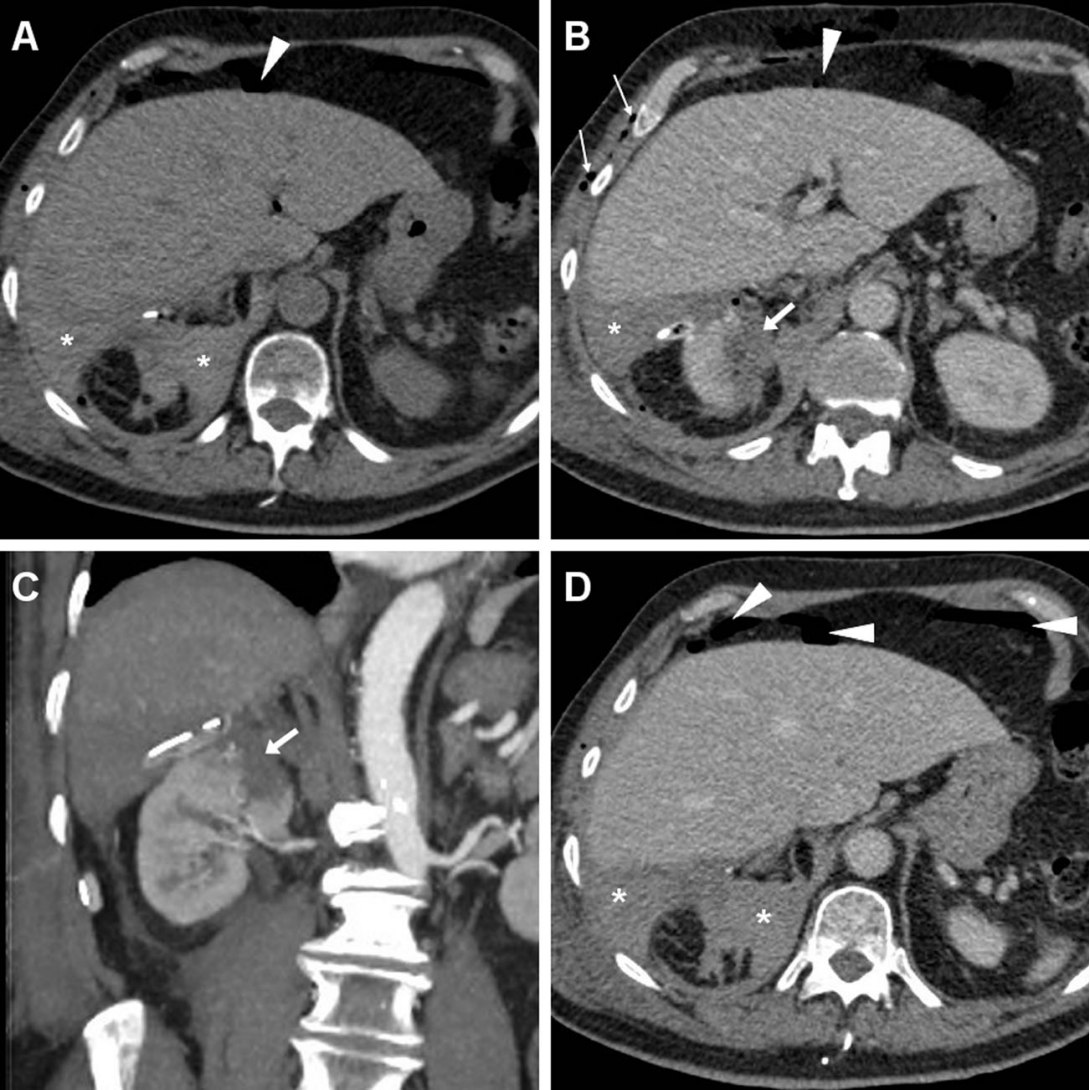
Haematoma in a 74-year-old man with diabetes, cardiac pacemaker, and progressive postoperative blood loss after L-PN. Unenhanced (**a**) and post-contrast (**b**, **c**) Multidetector CT images showed residual intraperitoneal gas bubbles (*arrowheads*) and minimal emphysema of the abdominal wall (*thin arrows* in **b**). Hyperattenuating blood collection (*) with the distal end of the drainage tube was seen along the posterior aspect of the right liver lobe, abutting the upper right renal pole with the hypoenhancing resection site (*arrows* in **b**, **c**) without CM extravasation indicating active bleeding in the corticomedullary phase (**b**, **c**) or urine leak in the excretory phase (**d**). The patient recovered with transfusion support

**Fig. 5:**
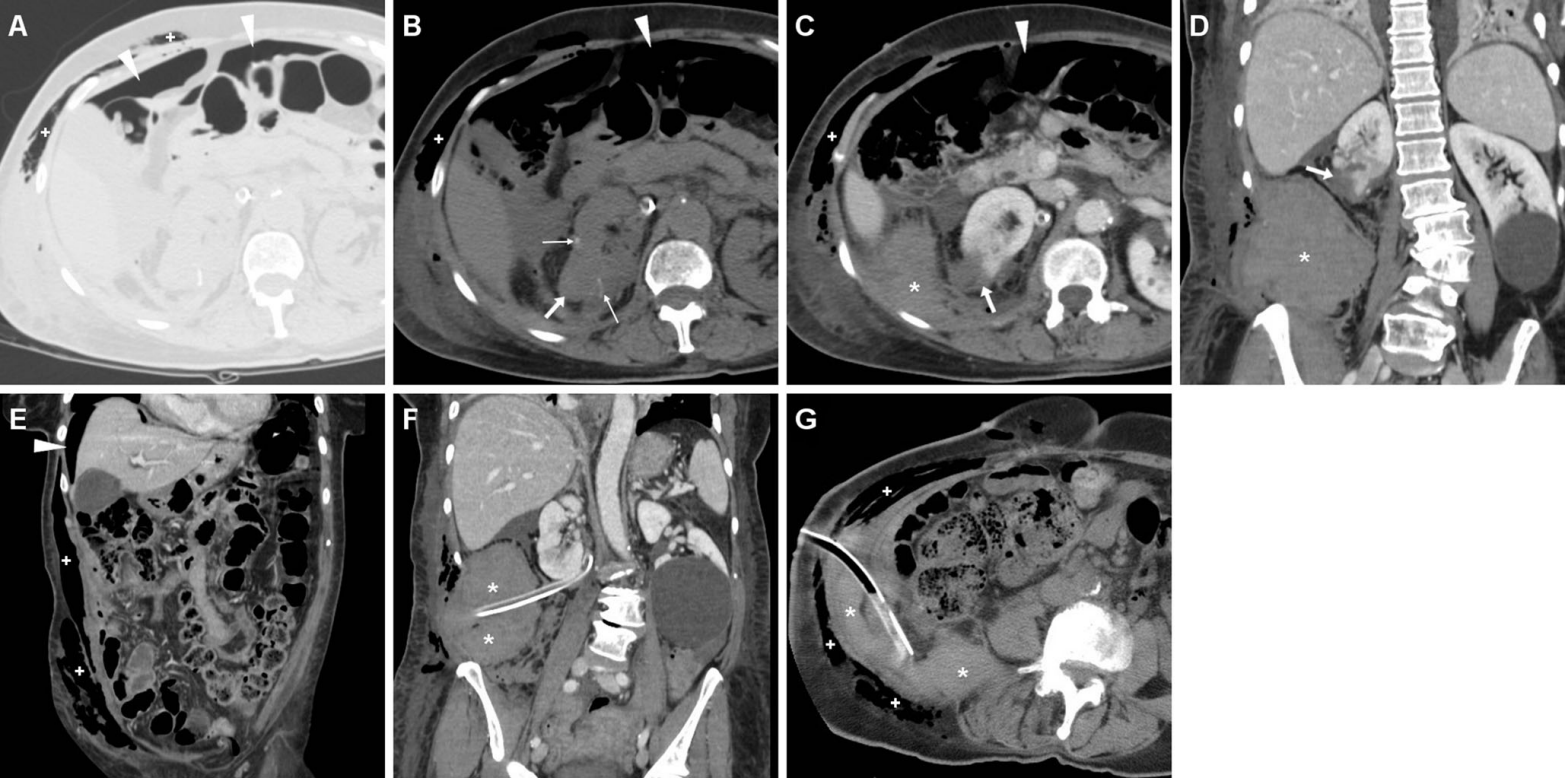
Two days after L-NSS for T1N0M0 RCC of the right kidney, the same patient as in Fig. 1a, b suffered from abdominal pain and progressive blood loss (nadir haemoglobin 11.5 g/dL), with stable vital signs. Urgent CT images viewed at lung window settings (**a**) showed moderate pneumoperitoneum (*arrowheads*) and subcutaneous emphysema of the abdominal wall (+). Unenhanced (**b**) and post-contrast (**c**–**f**) image showed the renal resection site (*arrows* in **b**–**d**) with sutures (*thin arrows*), a sizeable haematoma centred in the posterior para-renal space (*) causing kidney displacement, without active bleeding. Coronal CT reformation (**f**) confirmed massive subcutaneous emphysema (+) and pneumoperitoneum (*arrowhead*). Following conservative treatment and drainage tube repositioning, repeated CT 5 days later (**g**) showed persistent subcutaneous emphysema (+), decreased retroperitoneal blood collection (*)

Subcutaneous emphysema (Figs. 4, 5) is a common finding after laparoscopic surgery, and results from prolonged insufflation with the Trocar displaced into the abdominal wall. Emphysema is always non-dependent and most prominent immediately after surgery, and should not be misinterpreted as necrotising fasciitis, which usually occurs later and is associated with erythema, foul-smelling drainage from the wound, fever and pain [3].

In patients operated on through a TP laparoscopic approach, air-fluid levels of the small bowel consistent with adynamic ileus and minimal or moderate pneumoperitoneum (Figs. 4, 5) are commonly observed during the early postoperative period. If unknown, the type of laparoscopic access used may be guessed by searching for port access sites, in either the anterior ipsilateral abdomen (TP) or flank (retroperitoneal) [3]. Owing to the small incisions and the rapid absorption of CO_2_ from the perfusion gas, relative to that of room air, variable amounts of residual intraperitoneal free air are commonly observed following laparoscopy (in at least one-third of patients) within the first 3 days, and may sometimes last up to 9 days after surgery. However, postoperative pneumoperitoneum after laparoscopy is generally more limited than with open surgery, and decreases on serial imaging. Conversely, persistent or increasing intra-abdominal gas should raise concern for hollow viscus injury [3, 50, 51].

## Haemorrhagic and vascular complications

### Bleeding

Resulting from inadequate suturing or coagulation of transacted blood vessels, early postoperative haemorrhage with or without radiologically identifiable active bleeding represents the most common complication after L-NSS, with a reported incidence approaching 6–8 % of procedures. Blood transfusions are required in 5–21 % of patients and constitute a Clavien grade II complication [6–12].

**Fig. 6:**
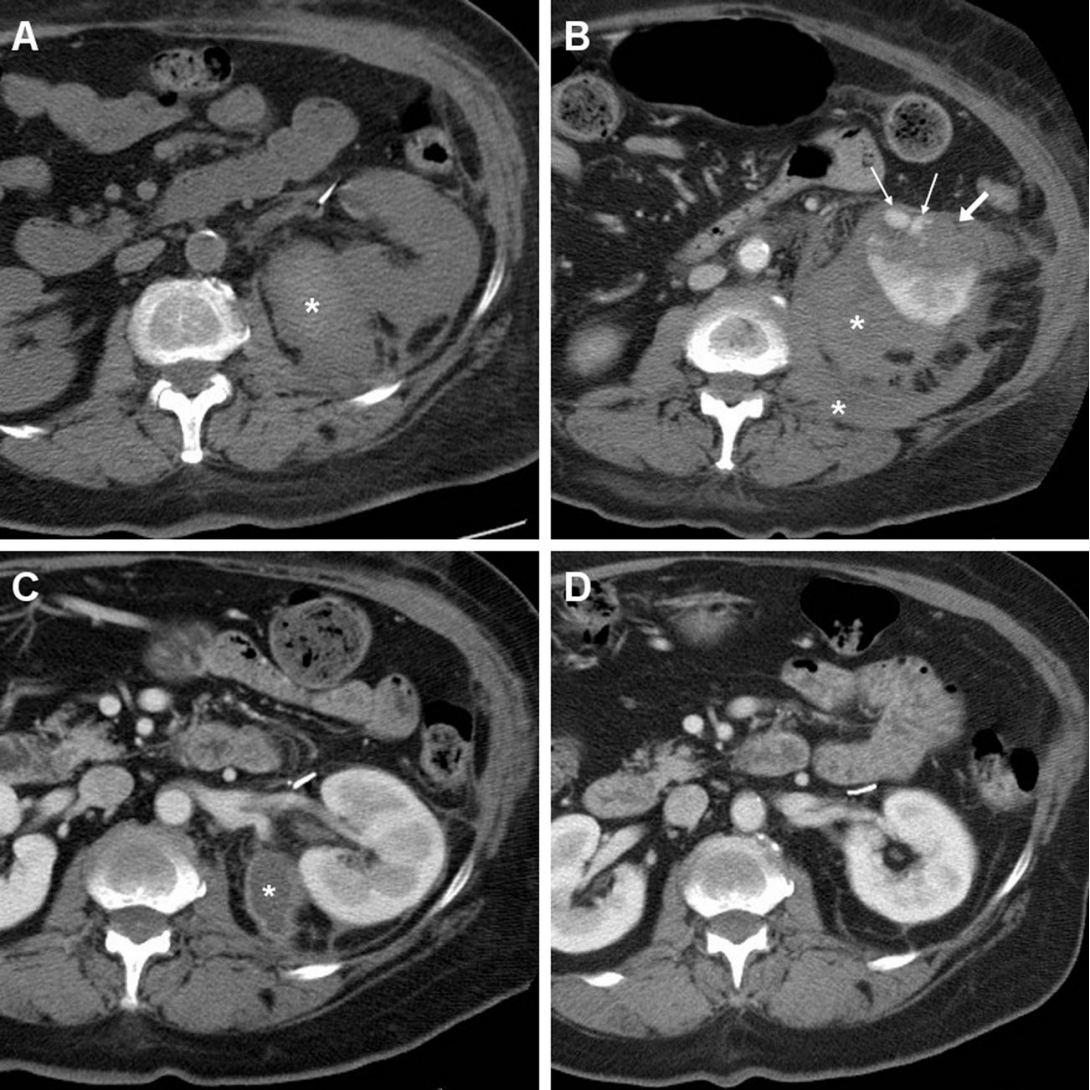
Abdominal pain and hypotension in a 62-year–old woman 24 h after laparoscopic enucleation of an early-stage RCC. Unenhanced CT acquisition (**a**) showed renal displacement by hyperattenuating medial peri-renal and posterior para-renal haematoma (*). Post-contrast corticomedullary-phase images (**b**) depicted foci of active extravasation (*thin arrows*) near the site of resection (*arrow*) at the resected inferior renal pole. The haematoma (*) become progressively liquefied and demarcated during CT follow-up (**c**) and eventually resolved 1 year later (**d**). (Partially reprinted with permission from Tonolini et al. [52])

As mentioned above, minimal or moderate degrees of peri- and para-renal blood are generally apparent after L-PN and should not be reported as abnormal. However, intra-luminal blood is closely related to gross haematuria and is identified on unenhanced CT scans as hyperattenuating content of the pelvicalyceal system (Fig. 3). In our experience, clinically significant iatrogenic haematomas after L-NSS appear as hyperattenuating collections compared to the renal parenchyma, which measure between 45 and 90 Hounsfield units (HU) depending on their stage, and mostly occupy the peri- and para-renal spaces (Figs. 4, 5, 6). Indicated by strongly hyperattenuating (85–370 HU, isodense to enhanced arterial vessels) foci corresponding to CM extravasation in the vicinity of the SSR, active bleeding at CT (Fig. 6) indicates a high risk of failure of conservative management and thus represents the strongest indication for interventional or surgical treatment. In addition, multidetector CT provides consistent follow-up of conservatively treated lesions (Figs. 5, 6) showing progressive demarcation, size reduction and decreasing attenuation of haematomas [22–25, 44, 52].

### Renal vascular complications

According to the EAU guidelines, iatrogenic renal vascular injuries (IRVI) rank among the rarest (less than 1 % overall), yet most feared complications after percutaneous biopsy, nephrostomy, nephroureterolithotomy, renal artery angioplasty or stenting, and NSS. The IRVI spectrum encompasses arterio-venous fistulas (AVFs), renal pseudoaneurysms (R-PA), vascular thrombosis and renal infarction. IRVIs can lead to significant morbidity, including massive haemorrhage, life-threatening haematuria, need for nephrectomy or deterioration of renal function [24, 32, 37].

Renal artery and intra-parenchymal pseudoaneurysms occur after 0.43–1.3 % of L-NSS procedures, and result from partial or complete injury to an intra-renal artery at the SSR, the main renal artery or one of its main branches. Specifically, R-PA may form when the combined effect of hypotension, coagulation and pressure from the adjacent structures leads to temporary cessation of the bleeding, followed by recanalisation from clot degradation. R-PAs may sometimes grow, become unstable from restorated blood flow, and eventually erode into the pelvicalyceal system or the surrounding perinephric tissues. Most cases are diagnosed during the first 2–3 weeks after surgery, occasionally after a few months’ delay. Diagnosing R-PA requires a high level of suspicion, since clinical manifestations are often vague or non-specific such as flank pain, gross haematuria, dizziness and fever [11, 32, 53, 54].

**Fig. 7:**
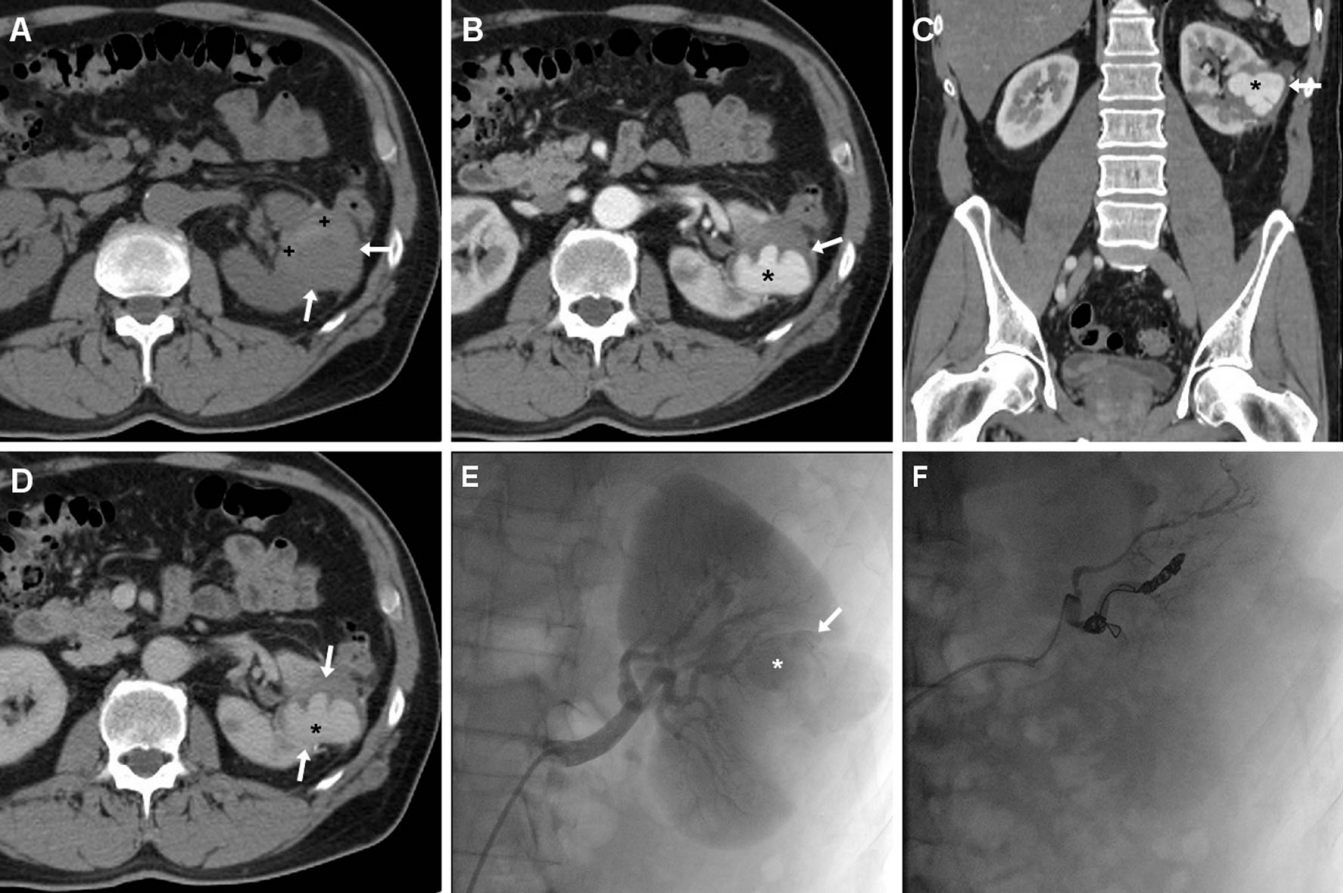
Postoperative gross haematuria in a 55-year-old man 5 weeks after enucleation of a left-sided, 3.7-cm, pT1a clear-cell RCC including the calyceal opening. Unenhanced CT images revealed a 4.5-cm, inhomogeneous, roundish renal mass (*arrowheads*) with non-dependent hyperattenuating material (+), suggesting thrombus. After intravenous contrast, corticomedullary (**b**, **c**) and nephrographic (**d**) CT images showed perfusion (*) synchronous to arterial vessels of most of the same lesion, consistent with pseudoaneurysm. Renal angiography (**e**) confirmed roundish lesion (*arrow*) occupied by haemorrhagic focus (*) from the distal portion of the anteromedial branch, corresponding to the site of recent tumour excision. Super selective catheterisation and embolisation with coils (**f**) allowed the bleeding to be stopped

Ultrasound may detect R-PA as a cystic mass or liquefied haematoma, with the characteristic “to-and-fro” internal flow at colour-Doppler sonography. On unenhanced CT images, a haematoma is commonly seen near to the surgical site, often associated with hyperattenuating blood clots in the renal pelvis, but the pseudoaneurysm itself is usually not visible. Conversely, arterial-phase CT-angiography acquisition shows a roundish, well-circumscribed lesion (usually measuring 1–3 cm) with contained arterial phase enhancement that appears isodense to the adjacent arterial vessels and becomes isoattenuating relative to the blood pool during the nephrographic phases (Fig. 7). Coronal CT-angiographic maximum intensity projection (MIP) reconstructions allow visualisation of the R-PA with its relationship to the renal vasculature. A R-PA may be missed in the excretory phase, owing to CM wash-out from the cavity [22–25, 44, 53, 55].

**Fig. 8:**
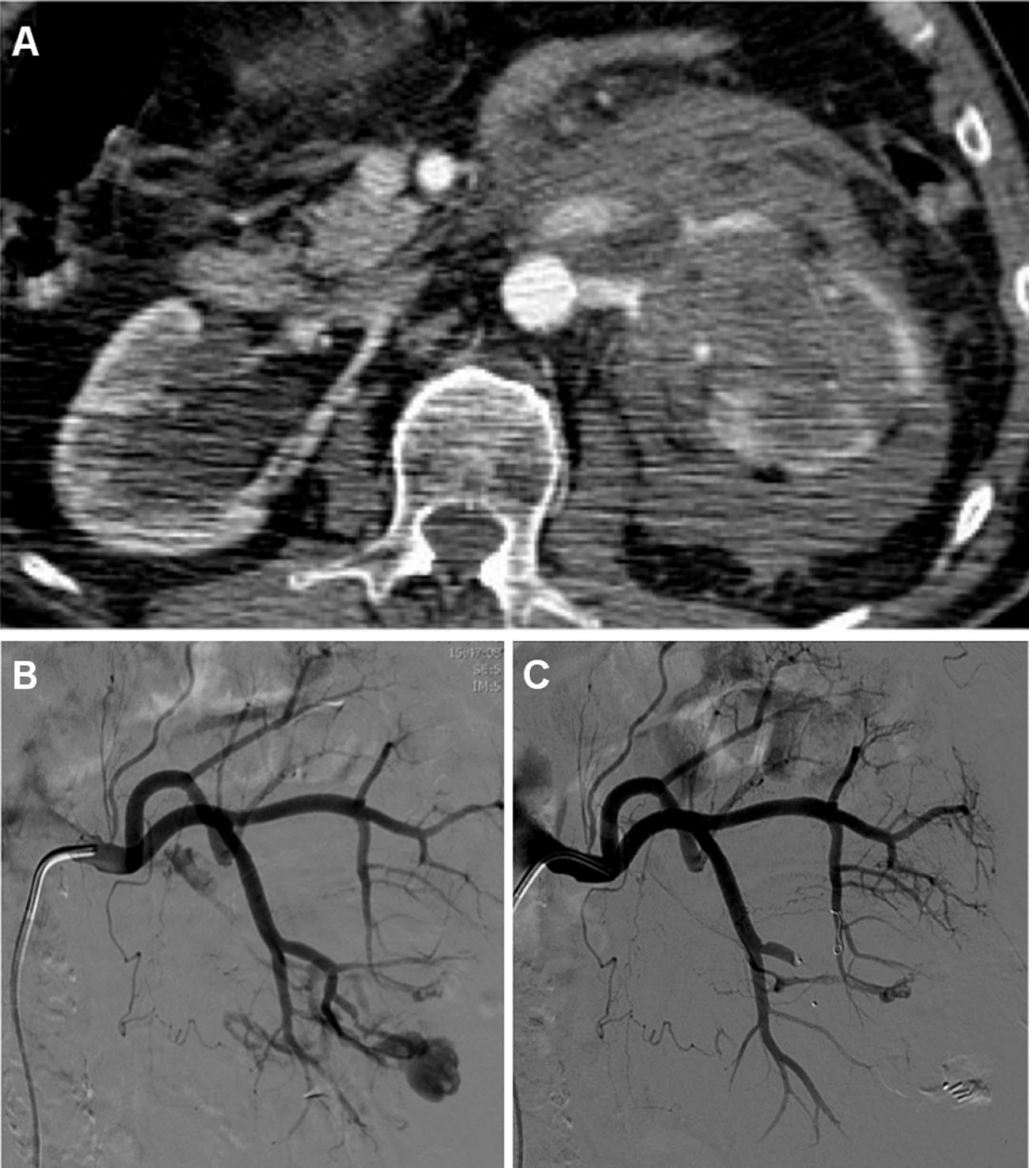
Progressive blood loss, haematuria and ipsilateral flank pain in a 75-year-old man 3 days following enucleation of a left-sided RCC. Arterial-phase CT acquisition (**a**) revealed a small pseudoaneurysm plus arterio-venous fistula suggested by early opacification of the left renal vein, which were confirmed by urgent angiography (**b**). After selective embolisation with a 4-mm Amplatzer vascular plug and 2-mm microcoil, repeated angiogram (**c**) demonstrated complete exclusion of both lesions

AVFs are most commonly iatrogenic rather than congenital; they are indicated by macro- or micro-haematuria from rupture within the collecting system, and may cause variable degrees of blood loss. The CT hallmark of AVF includes tortuous arteries and early or simultaneous opacification of one or more intra-parenchymal arteries and veins during the corticomedullary phase (Fig. 8) [55, 56].

Occasionally, arterial clamping may injure the vessel intima, thus leading to thrombosis, infarction and atrophy. CT-angiography depicts arterial thrombosis as abrupt vessel cut-off, and renal infarction (Fig. 8) as a peripheral wedge-shaped non-enhancing area in the renal parenchyma, with a rim of enhancement at the periphery of the cortex (rim sign) due to preserved capsular blood flow [22, 24].

Selective angiography evaluates the lesion dynamically and allows planning for trans-arterial embolisation (TAE) and super-selective catheterisation [56]

### Treatment

**Fig. 9:**
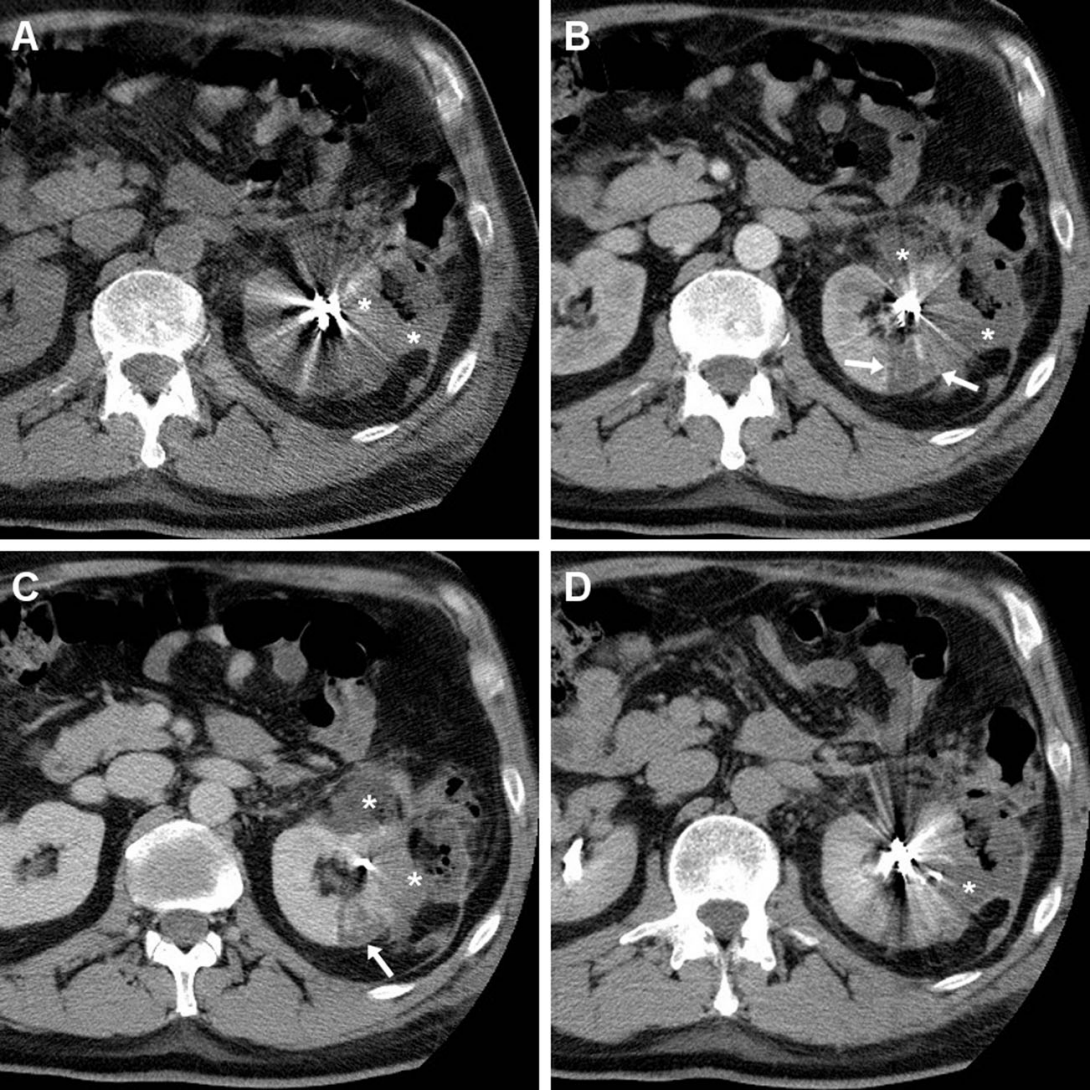
Post-embolisation status in a 53-year-old man with acute haemorrhage after L-PN. Selective embolisation of arterial branches with Embozene particles and metallic coils achieved bleeding control and prompt clinical improvement. Five days later, unenhanced (**a**), corticomedullary (**b**) and nephrographic (**c**) phase multidetector CT acquisitions showed strongly hyperdense coils at the renal sinus causing beam-hardening artefacts, some residual peri-renal blood (*) without persistence of active bleeding, and a wedge-shaped non-enhancing parenchymal area (*arrows*) consistent with focal infarction. Excretory (**d**) phase images excluded urine leak from the collecting system

In the past, severe bleeding and IRVIs required laparotomy. Currently, the therapeutic approach is increasingly conservative, provided that clinical and laboratory parameters remain stable and CT excludes active bleeding. Although haematomas and IRVI may heal spontaneously with conservative management, selective TAE (Figs. 7, 8, 9) is recommended as the established minimally invasive treatment of choice for life-threatening occurrences with persisting or massive bleeding, severe haematuria from communication to the pelvicalyceal system, or progressively deteriorating renal function. Notably, the need for interventional treatment such as TAE constitutes a major (Clavien grade III) complication. Arteriography confirms R-PA as an ovoid CM pool close to the SSR (Fig. 7). In patients with post-surgical IRVI, TAE using coils or other embolisation agents proved to be safe and effective treatment with a low rate of complications, rapid recovery and short hospital stay, and excellent (nearly 100 %) technical and clinical success rates, respectively corresponding to complete angiographic exclusion of bleeding and haemodynamic stabilisation without the need for blood transfusion. Super-selective embolisation, carried out as distally as possible is recommended to minimise parenchymal loss and avoid long-term impairment of renal function [32, 55, 56].

## Miscellaneous complications

### Postoperative infections

**Fig. 10:**
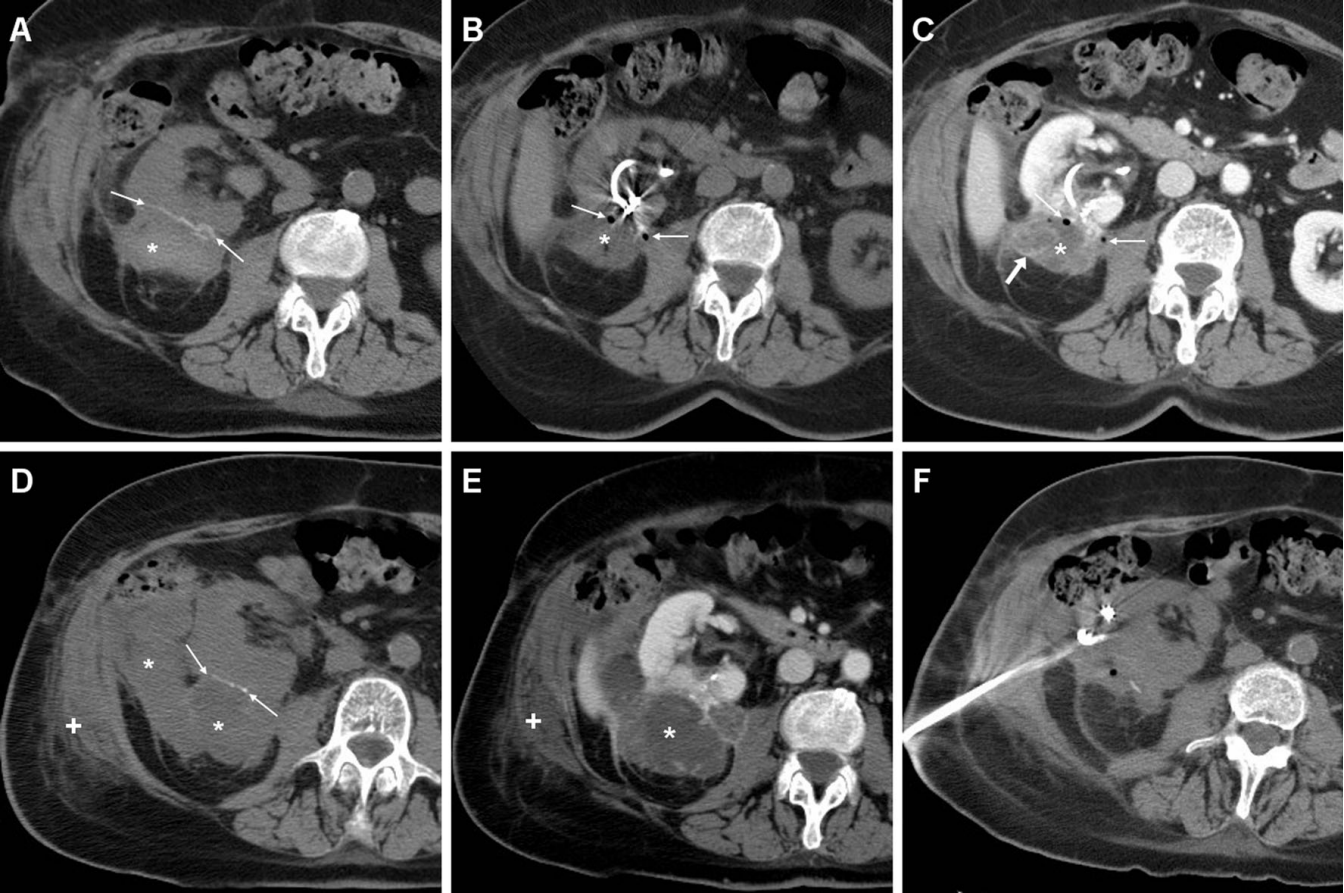
A 69-year-old woman suffering from persistent pain and haematuria 10 days after right renal tumour resection had evidence of confined peri-renal haematoma (*) abutting the site of resection indicated by sutures (*thin arrows*) on unenhanced (**a**) CT. After conservative treatment repeated CT (**b**, **c**—note positioning of ureteral stent) the collection (*) showed decreased attenuation, appearance of non-dependent gas bubbles (*thin arrows*) and rim-like enhancement (*arrow* in **c**) suggesting infection. Abscessualisation (*) was confirmed by significant size increase despite antibiotic treatment at follow-up CT (**d**, **e**) with persistent peripheral enhancement, by appearance of oedematous inflammatory thickening of the ipsilateral abdominal wall (+), and was relieved by percutaneous drainage (**f**)

Occasionally, fluid collections (seroma, haematoma or urinoma) forming close to the SSR after L-PN may become infected. Differentiation of non-infected collections from an abscess requires correlation of clinical signs and laboratory data with imaging appearances, mostly represented by enlarging intra- or peri-renal hypoattenuating collections with a peripheral, thickened enhancing capsule (Fig. 10). Alternatively, the residual renal parenchyma may show the characteristic “striated nephrogram” appearance consistent with pyelonephritis [22–25, 57, 58].

### Urine leaks and urinomas

Surgical access to the collecting system is necessary to ensure an adequate margin of resection for tumours extending deeply into the renal parenchyma: if the subsequent pelvicalyceal repair is not watertight, urine may leak into the surgical bed leading to a peri-renal urinoma or collection of a mixture of blood and urine. Urine leakage has been estimated to occur in 1.3-3.6 % of L-PN interventions [6–12].

**Fig. 11:**
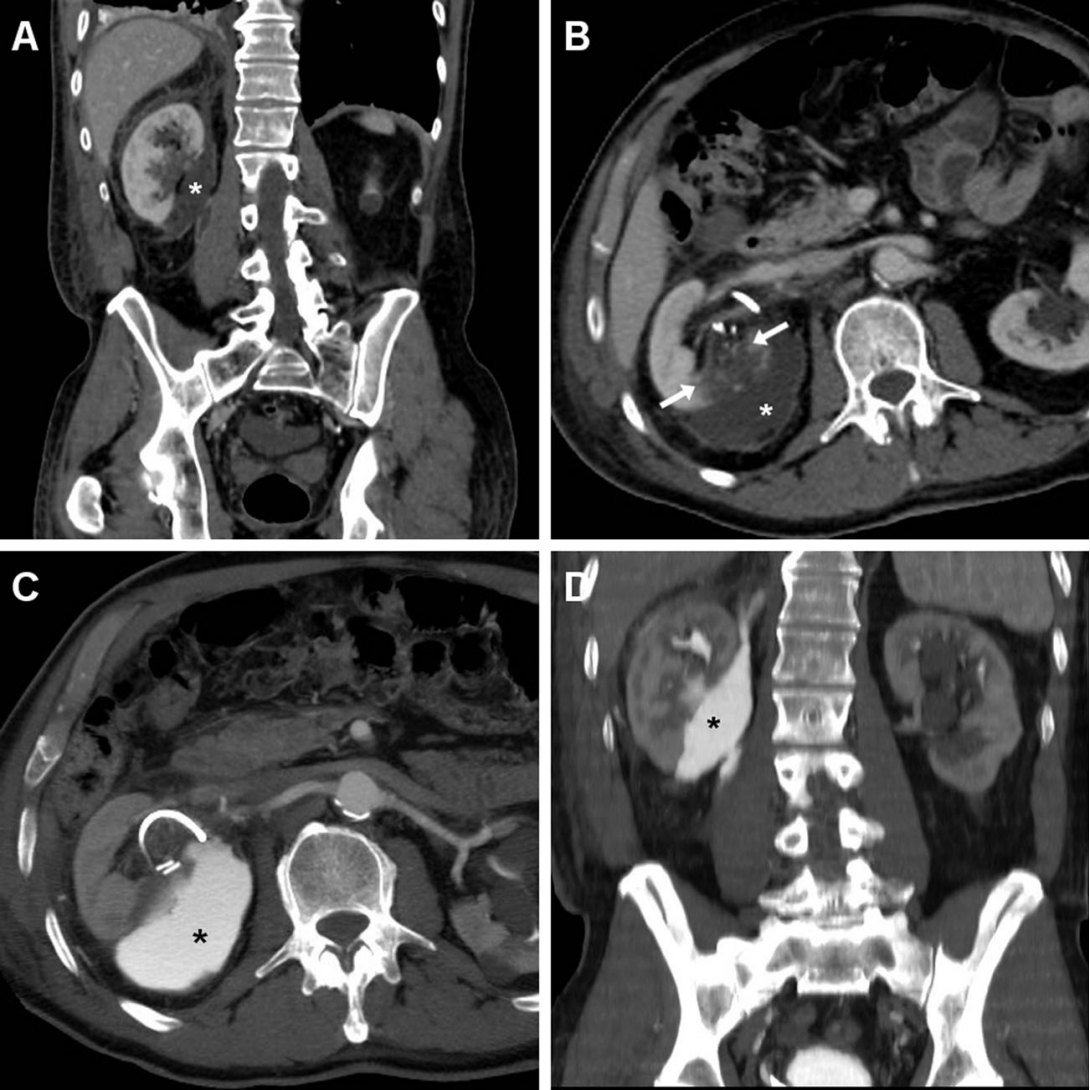
Peri-renal urinoma in a 73-year-old man investigated with multidetector CT after laparoscopic resection of right-sided renal tumour because of progressive blood loss and acute flank pain. Contrast-enhanced coronal (**a**) and axial (**b**) images showed a medial peri-renal fluid collection (*), ureteral stent in place and clips at the renal hilum, and a 2.5-cm hypoenhancing portion of the posterior renal cortex (*between arrows*) corresponding to the resection site. Forty-eight hours later, follow-up split-bolus CT-urography (**c**, **d**) showed minimally enlarged collection filled with enhanced urine consistent with urinoma from injured renal pelvis. Postoperative course included prolonged ureteral stenting and blood transfusions. (Partially reprinted with permission from Tonolini et al. [52])

The characteristic appearance of leakage seen on CT relies on identification of CM-opacified urine (80–200 HU) extravasated from the collecting system into the peri-renal space, visualised on excretory phase acquisitions (Fig. 11). Urinomas appear as more or less homogeneous collections, with progressive opacification over repeated delayed acquisitions [22–25, 49, 52].

Most urine leaks resolve spontaneously over time and are generally managed conservatively with ureteral stenting, Foley catheter or percutaneous nephrostomy. Endourological fulguration and percutaneous imaging-guided drainage may be required to treat persistent leaks and urinomas, respectively [29, 49].

## Conclusions

In most patients with suspected postoperative complications after L-NSS, urgent multidetector CT imaging allows detection of intraluminal and peri-renal haemorrhage, active bleeding, vascular injuries, extravasated urine and infections. Therefore, CT findings usually provide a consistent basis for assessment of the severity of injury and a correct choice between conservative treatment, TAE, repeated surgery, nephrostomy and/or ureteral stenting [44, 49].
